# Tumor-associated macrophages and lineage plasticity in prostate cancer: from established myeloid programs to emerging spatial hypotheses

**DOI:** 10.3389/fimmu.2026.1853196

**Published:** 2026-07-13

**Authors:** Jia Li, Jinling Li, Yuechao Zhao, Yuanqi Yu, Xiaolu Cui

**Affiliations:** 1 Phlebotomy Center, First Hospital of China Medical University, Shenyang, Liaoning, China; 2 Department of Endoscopy, First Hospital of China Medical University, Shenyang, Liaoning, China; 3 Department of Pediatrics, First Hospital of China Medical University, Shenyang, Liaoning, China; 4 Department of Urology, First Hospital of China Medical University, Shenyang, Liaoning, China

**Keywords:** prostate cancer, tumor-associated macrophages, lineage plasticity, tumor microenvironment, spatial transcriptomics, androgen receptor signaling inhibitors, therapeutic resistance

## Abstract

Androgen receptor signaling inhibitors (ARSIs) have transformed the treatment of advanced prostate cancer, yet durable responses are limited by the emergence of therapy-resistant disease states, including neuroendocrine prostate cancer (NEPC) and double-negative prostate cancer (DNPC). Lineage plasticity has traditionally been studied from a tumor-cell-intrinsic perspective, but single-cell and spatial studies increasingly indicate that the tumor microenvironment, particularly tumor-associated macrophages (TAMs), may influence tumor-cell state transitions, immune exclusion, and therapeutic resistance. In this review, we synthesize established and emerging evidence linking TAM heterogeneity to prostate cancer lineage plasticity. We first summarize independently supported myeloid programs, including SPP1+/TREM2+ macrophage states and TAM-derived pathways such as IL-6/STAT3, TGF-beta, NF-kappaB, CXCL12/CXCR4, and adenosine signaling. We then discuss PLAC8+ TAMs, TNFAIP8L2, and PLAC8+ TAM/CXCL12+ iCAF/CD8+ TRM spatial aggregates as an emerging, hypothesis-generating framework that may be associated with ARSI-induced DNPC-like remodeling. Importantly, we explicitly distinguish spatial and transcriptomic associations from experimentally proven causal mechanisms. The proposed TNFAIP8L2-integrin/PI3K-Akt/beta-catenin/FOSL1-HMGA1 cascade is therefore presented as a working model that requires direct biochemical, genetic, and *in vivo* validation. Finally, we outline an evidence-aware translational roadmap for TAM-directed therapy, emphasizing independent cohort validation, protein-level spatial confirmation, functional perturbation, and biomarker-guided clinical testing.

## Graphical Abstract

**Figure:**
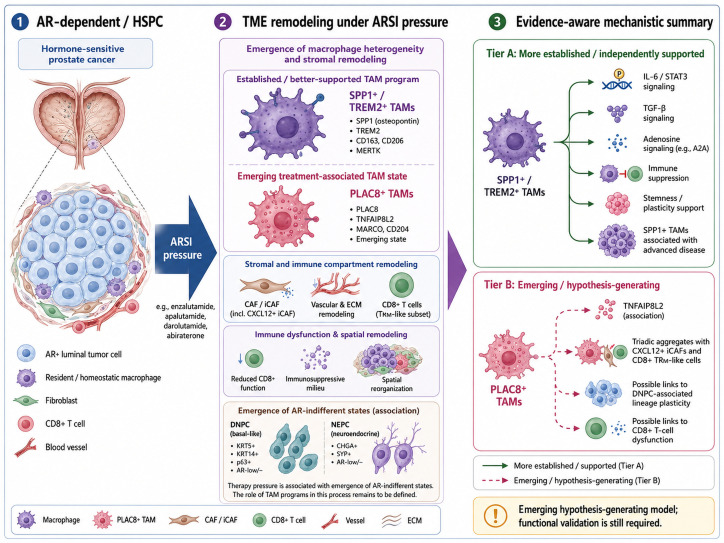
Evidence-aware overview of TAM heterogeneity and prostate cancer lineage plasticity under ARSI pressure. Under androgen receptor signaling inhibitor (ARSI) pressure, the prostate tumor ecosystem may undergo epithelial, stromal, vascular, and immune remodeling. The graphical abstract separates better-supported TAM programs, such as SPP1+/TREM2+ macrophage states and established cytokine or adenosine pathways, from emerging PLAC8+ TAM/TNFAIP8L2-associated hypotheses. Solid arrows indicate relatively better-supported associations or mechanisms, whereas dashed arrows indicate hypothesis-generating links, including possible relationships between PLAC8+ TAMs, CXCL12+ iCAFs, CD8+ TRM-like cells, and DNPC-associated plasticity. This evidence-aware framework is intended to guide interpretation and validation rather than to imply that PLAC8+ TAMs are already proven causal drivers of lineage conversion.

## 1 Introduction

Prostate cancer remains a major cause of cancer morbidity and mortality among men worldwide (1). Although androgen deprivation therapy (ADT) and subsequent androgen receptor signaling inhibitors (ARSIs) substantially improve outcomes in advanced disease, resistance commonly develops (2, 3). A clinically challenging manifestation of resistance is the emergence of AR-indifferent disease states, including double-negative prostate cancer (DNPC) and neuroendocrine prostate cancer (NEPC) (4–6). These phenotypes are generally less responsive to conventional hormonal manipulation and have shown limited benefit from immune checkpoint blockade in unselected advanced prostate cancer cohorts; however, recent work also indicates that AR inhibition can alter T-cell function and MHC class I antigen presentation, suggesting that immunotherapy resistance should be framed as context-dependent rather than absolute (7–9).

The molecular basis of prostate cancer lineage plasticity has often been investigated from a tumor-cell-intrinsic perspective, with studies identifying transcriptional, epigenetic, and genomic regulators such as SOX2, TP63, RB1/TP53 loss, chromatin remodeling factors, and the FOSL1-HMGA1 axis (10–13). However, this cell-autonomous framework is incomplete. The tumor microenvironment (TME) can shape tumor-cell fate through paracrine signaling, stromal remodeling, metabolic constraints, immune selection, and direct cell-cell interactions (14, 15). Among TME components, tumor-associated macrophages (TAMs) are particularly relevant because they are abundant, plastic, and capable of integrating inflammatory, metabolic, stromal, and therapeutic cues.

Single-cell transcriptomic technologies have substantially revised the traditional M1/M2 view of macrophage biology (16, 17). Rather than existing as two stable polarization states, TAMs occupy a continuum of transcriptional and functional programs that vary by tissue context, tumor stage, metastatic site, and treatment exposure (18, 19). In prostate cancer, multiple studies have identified myeloid populations associated with immune suppression, stromal remodeling, metastasis, therapy resistance, and adverse clinical outcomes (7).

Spatial transcriptomic and proteomic technologies add an essential dimension by mapping these TAM states within native tissue architecture (20). Such approaches can reveal non-random neighborhoods involving macrophages, cancer-associated fibroblasts, lymphocytes, and malignant epithelial cells (21, 22). However, spatial proximity is correlative and cannot, by itself, establish receptor-ligand engagement, protein secretion, or functional causality. This distinction is especially important for newly proposed spatial structures such as PLAC8+ TAM/CXCL12+ iCAF/CD8+ TRM triadic aggregates (11).

Recent prostate cancer single-cell and spatial atlases provide the broader context for interpreting TAM-associated lineage plasticity (23–32). These studies demonstrate extensive heterogeneity in epithelial, stromal, and immune compartments and support the concept that lineage plasticity occurs within an evolving tissue ecosystem. They also underscore the need to distinguish robustly replicated macrophage programs, such as SPP1+/TREM2+ states, from newer treatment-associated states, such as PLAC8+ TAMs, whose independent validation remains limited.

**Figure 1:**
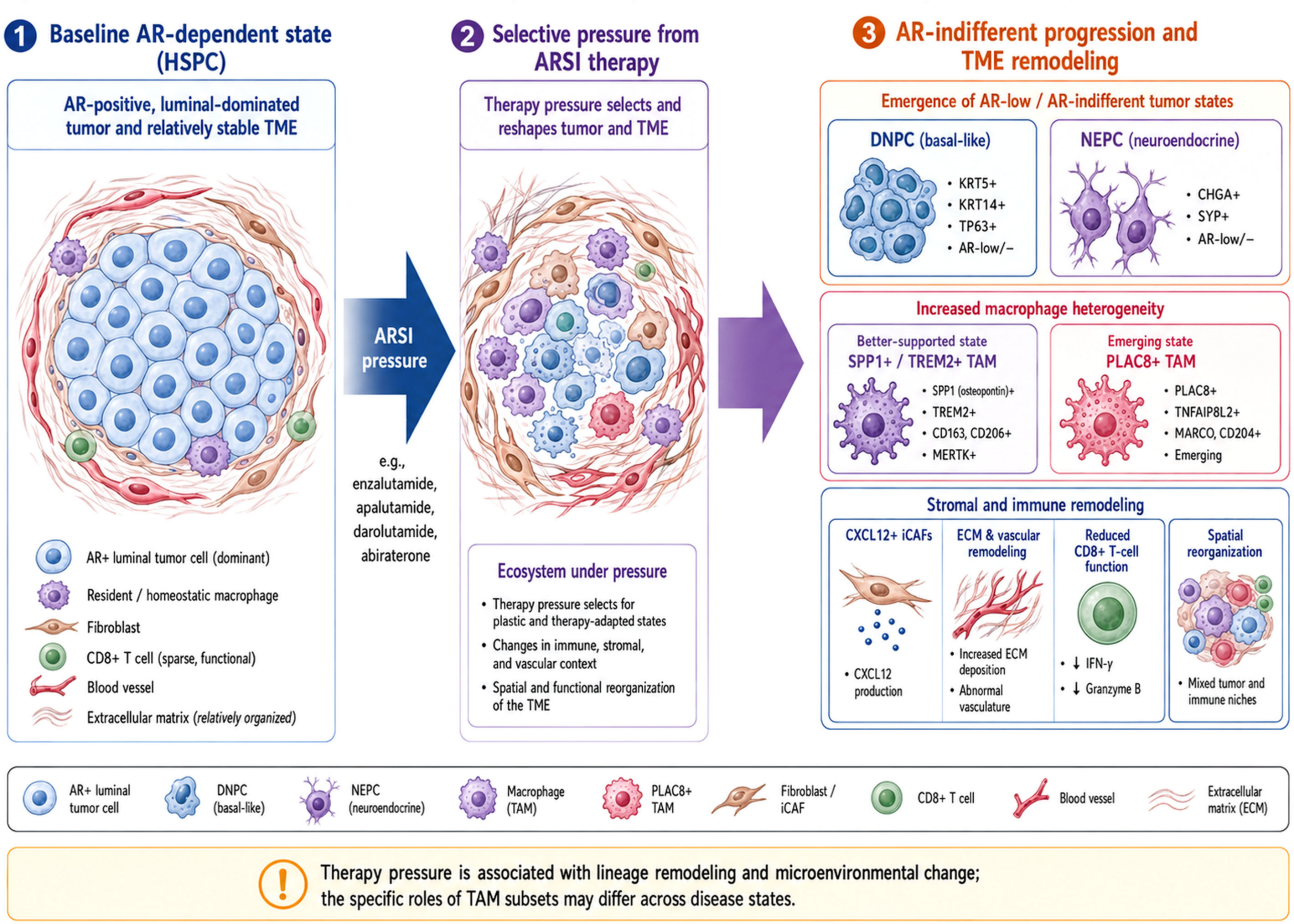
Conceptual overview of prostate cancer lineage remodeling and microenvironmental changes under ARSI pressure. The figure summarizes a staged, non-deterministic model of therapy-associated ecosystem remodeling. In the baseline AR-dependent/hormone-sensitive state, tumors are predominantly composed of AR-positive luminal cells within a relatively organized stromal and immune context. ARSI therapy imposes selective pressure that may enrich therapy-adapted epithelial states and reshape the immune, stromal, extracellular matrix, and vascular compartments. In advanced disease, AR-low or AR-indifferent phenotypes, including DNPC-like and NEPC-like states, may emerge together with increased macrophage heterogeneity, CXCL12+ iCAF activity, reduced CD8+ T-cell function, and spatial reorganization. The figure emphasizes association and co-evolution rather than a single TAM subset as a proven causal driver of lineage remodeling.

This review therefore adopts an evidence-aware framework. **Figure 1** summarizes the revised conceptual model, in which ARSI-associated epithelial remodeling and TAM-state changes are presented as co-evolving features of disease progression rather than as a single linear causal pathway. For clarity, we distinguish established findings supported by multiple independent studies or functional data from emerging observations supported by limited prostate cancer cohorts, spatial associations inferred from tissue proximity, and speculative working hypotheses that require direct perturbational validation.

## 2 TAM heterogeneity in prostate cancer: from established myeloid programs to emerging treatment-associated states

TAMs represent a major immune component of the prostate tumor microenvironment and are increasingly recognized as heterogeneous, context-dependent populations rather than a uniform immunosuppressive compartment (33–35). The historical M1/M2 framework remains useful as a simplified vocabulary, but it is insufficient to describe macrophage states observed in human tumors, where ontogeny, tissue localization, hypoxia, stromal contact, metabolic stress, and therapy exposure can all influence macrophage phenotype.

scRNA-seq and spatial approaches have shown that TAMs exist as a spectrum of functional states with distinct transcriptional programs, metabolic features, and spatial distributions (36–38). In prostate cancer, this heterogeneity is clinically relevant because different myeloid states have been linked to tumor progression, metastatic adaptation, immune suppression, and resistance to systemic therapy.

### 2.1 Beyond the M1/M2 framework and technical considerations

Single-cell technologies enable unbiased discovery of macrophage states without relying on *a priori* surface-marker definitions (39, 40). However, marker-based terminology should be interpreted cautiously: SPP1, TREM2, APOE, C1QC, PLAC8, HLA-DQB2, CD163, and CD206 mark transcriptional states rather than immutable macrophage lineages. These states may vary across platforms, sampling sites, disease stages, and treatment contexts.

Cross-tumor studies have identified recurrent TAM programs such as SPP1+, TREM2+, APOE+, and C1QC+ macrophages (16, 41–46). These observations support the broader concept of TAM heterogeneity, but prostate cancer-specific evidence is required before assigning disease-specific functions to any subset. Accordingly, the following section prioritizes prostate cancer single-cell and spatial studies rather than extrapolating directly from other tumor types.

### 2.2 Independently supported TAM programs in prostate cancer

Among prostate cancer TAM states, SPP1+/TREM2+ and related macrophage programs currently have the strongest independent support. Multiple prostate cancer single-cell and spatial studies have identified SPP1-expressing macrophages in advanced, metastatic, or immune-suppressed tumor ecosystems (7, 30, 47). These cells are often associated with extracellular matrix remodeling, angiogenesis, hypoxia, lipid metabolism, impaired antigen presentation, and adverse clinical outcomes.

Integrated single-cell and spatial analyses have identified SPP1+/TREM2+ TAM populations enriched in metastatic or aggressive prostate cancer regions (47, 48). Spatial localization within tumor cores, invasive margins, or metastatic niches may place these macrophages in proximity to malignant epithelial cells and stromal remodeling programs. These associations support a pro-tumorigenic role, although the precise functional contribution of each SPP1+ TAM state may vary by disease stage and tissue context.

Across the disease continuum, SPP1hi TAMs appear to increase during progression toward mCRPC and may contribute to immune suppression through hypoxia-adenosine programs and reduced responsiveness to immune checkpoint blockade (7, 49). These findings provide an important comparator for newer TAM states because they are supported by independent prostate cancer datasets and, in some contexts, by functional studies.

Cross-cancer preclinical work and prostate cancer-specific studies suggest that SPP1+ macrophage programs can be linked to immune suppression, stromal crosstalk, and immunotherapy resistance (7, 50). These data support the therapeutic relevance of SPP1+ TAMs, but their role should be interpreted within a broader ecosystem that includes stromal cells, tumor epithelial states, and other suppressive myeloid populations.

Beyond SPP1-expressing populations, prostate cancer atlases have identified additional myeloid, stromal, and epithelial states associated with immune suppression, exhausted T-cell phenotypes, club-like or chemokine-enriched epithelial programs, and lineage-plastic tumor-cell modules (23–25, 30–32). These broader atlases provide important context and prevent over-attribution of DNPC-associated plasticity to a single macrophage subset.

Collectively, these studies support SPP1+/TREM2+ TAMs as recurrent pro-tumor-associated programs in advanced prostate cancer. In contrast, the possibility that distinct AR-indifferent states such as DNPC involve additional treatment-associated macrophage programs remains an important but less mature hypothesis. Therefore, PLAC8+ TAMs are discussed below as an emerging state that may be associated with ARSI-treated and DNPC-like ecosystems, not as a necessary or independently established driver of lineage plasticity.

### 2.3 PLAC8+ TAMs as an emerging treatment-associated macrophage state

Prostate cancer TAM states can be described using marker combinations, but these markers should be interpreted as state-associated rather than lineage-defining. In this context, PLAC8+ TAMs have been proposed as a treatment-associated macrophage state enriched in ARSI-treated disease and linked to DNPC-like or basal-like tumor programs (11). HLA-DQB2 has been proposed as a candidate surface marker, whereas TNFAIP8L2 has emerged as a candidate secreted effector. However, these observations remain early and should be interpreted as an emerging association that requires independent validation at the transcript, protein, spatial, and functional levels.

Distinct TAM states may also exhibit different metabolic programs, including lipid metabolism, oxidative phosphorylation, xenobiotic metabolism, and hypoxia-related pathways (53–56). Spatial analyses suggest that SPP1+ and PLAC8+ TAMs may occupy different tissue neighborhoods (7, 11, 23, 30, 47). SPP1+ TAMs have been associated with hypoxic, invasive, metastatic, or stromal-remodeling regions, whereas PLAC8+ TAMs have been proposed to appear within therapy-associated spatial neighborhoods. These spatial patterns are informative but should be considered association-generating rather than proof of subset-specific biological functions.

### 2.4 TAM plasticity and dynamic evolution

An emerging concept in TAM biology is that macrophage states are not static entities but evolve in response to changing microenvironmental conditions and therapeutic pressure (35). ADT and ARSI can reshape the prostate tumor immune microenvironment (57–59), and reported PLAC8+ TAM enrichment after therapy raises the possibility that treatment pressure favors specific macrophage programs, either through monocyte recruitment, tissue-resident macrophage adaptation, or local expansion. Trajectory analyses further suggest that SPP1+ and PLAC8+ TAM states may follow different developmental routes, although lineage relationships remain unresolved without direct fate-mapping or longitudinal validation.

Temporal and spatial correlations between TAM states and tumor phenotypes are informative but should be interpreted cautiously (38). SPP1+ TAMs have been associated with neuroendocrine or advanced disease programs, whereas PLAC8+ TAMs have been linked to basal-like or DNPC-associated states in limited settings. These associations suggest that macrophage programs may accompany or reinforce lineage remodeling, but functional experiments are needed to determine whether macrophages initiate, maintain, or merely mark lineage transitions. This distinction is therapeutically important because selective targeting of one myeloid state may favor compensatory expansion of other suppressive populations; consequently, combination strategies should be guided by validated biomarkers rather than by subset labels alone.

Interactions between TAMs, rare epithelial states, CAFs, and age-related stromal programs add additional complexity. Club-like or chemokine-enriched gland signatures may influence myeloid recruitment, but these axes are peripheral to the PLAC8+ TAM/DNPC hypothesis and are discussed here only as broader context. Overall, single-cell and spatial studies have replaced the simplistic M1/M2 dichotomy with a more nuanced view of prostate cancer TAM heterogeneity: SPP1+/TREM2+ TAMs are relatively well supported across independent studies, whereas PLAC8+ TAMs represent a newer treatment-associated state that requires further validation. **Table 1** summarizes these TAM-related concepts with attention to evidence maturity and proposed therapeutic relevance. A more detailed overview of representative TAM, myeloid, and stromal programs, including marker combinations, biological context, evidence maturity, and selected supporting references, is provided in **Supplementary Table S1**.

**Table 1:** Evidence hierarchy for TAM-associated concepts in prostate cancer lineage plasticity.

Concept/axis	Evidence level	Current support	Interpretation and therapeutic implication	Representative refs/caveats
PLAC8+ TAMs/TNFAIP8L2 axis	Emerging/hypothesis-generating	Reported after ADT/ARSI exposure; candidate markers include PLAC8, HLA-DQB2 and TNFAIP8L2; independent validation remains limited.	Potential treatment-associated macrophage state spatially associated with DNPC-like regions; TNFAIP8L2-centered signaling is a working model; direct causal effects on CD8+ TRMs or tumor-cell transdifferentiation remain unproven.	(11, 51, 52, 60–62); HLA-DQB2 depletion and TNFAIP8L2 neutralization are preclinical/speculative.
SPP1+/TREM2+ TAMs	Relatively established in prostate cancer	Multiple independent prostate cancer single-cell and spatial studies; associated with mCRPC, metastasis, hypoxia, ECM remodeling and immunosuppression.	Independently supported comparator myeloid program; may influence immune suppression through adenosine and related pathways; supports testing of adenosine/A2AR, SPP1, or TREM2 approaches in selected contexts.	(7, 30, 47–50, 63, 64)
Prostate cancer lineage-plastic tumor states	Established tumor-cell phenomenon; TME links emerging	Multiple bulk, single-cell, and spatial datasets define AR-low, NEPC, DNPC, club-like, and lineage-plastic tumor programs.	Provides the tumor-cell context in which TAM-associated hypotheses should be tested; therapeutic implications depend on whether myeloid/stromal cues are causal amplifiers or bystanders.	(4, 5, 10, 11, 23, 25, 31, 32, 65, 66)
IL-6, TGF-beta, CXCL12 and adenosine pathways	Established or partly validated	Functional and translational literature supports roles in stemness, immune suppression, T-cell exclusion and therapy resistance.	More mature mechanistic foundation than a single TNFAIP8L2-centered model; useful for combination hypotheses with ARSI, ICB, A2AR blockade, CXCR4 blockade, or pathway-specific validation.	(8, 9, 63, 67–73)
CSF1R-dependent macrophage programs	Established general TAM pathway; limited subset specificity	Broad macrophage survival and recruitment pathway; clinical activity in prostate cancer has been modest.	Useful background strategy but not a precise marker of lineage-plasticity-associated TAM states; broad depletion may cause toxicity and compensatory myeloid expansion.	(49, 74–77)
PMN-MDSCs and compensatory myeloid programs	Established suppressive myeloid population; not a TAM subset	May expand after TAM-directed pressure and contribute to resistance to macrophage-targeted therapy.	Important confounder and potential escape route when interpreting TAM-targeted interventions; CXCR2/IL-8 blockade may be considered in combination settings but requires validation.	(78–80)

## 3 TAM-derived molecular signals in prostate cancer lineage plasticity

The preceding section highlighted the heterogeneity of prostate cancer TAMs. Here, we discuss molecular pathways through which macrophages may influence tumor-cell plasticity, emphasizing mechanisms with stronger experimental support before considering TNFAIP8L2-centered models as emerging hypotheses.

### 3.1 Multimodal paradigms of TAM-tumor cell communication

TAMs engage tumor cells through multiple complementary mechanisms that collectively shape the tumor microenvironment and influence lineage identity. Paracrine signaling represents the most extensively characterized mode of communication, wherein TAM-derived soluble factors—including cytokines, chemokines, growth factors, and lipid mediators—diffuse through the extracellular space to engage cognate receptors on tumor cells (81, 82). This mode enables spatially distributed signaling over distances ranging from tens to hundreds of micrometers, allowing TAMs to profoundly influence tumor cell behavior, survival, and plasticity even when not in direct physical contact (83).

Beyond soluble factors, direct receptor-ligand interactions can mediate juxtacrine communication between macrophages, lymphocytes, stromal cells, and tumor cells (84). In the case of TNFAIP8L2, the identity of a functional receptor on prostate cancer cells remains insufficiently established. Therefore, any proposed TNFAIP8L2-integrin-related signaling cascade should be presented as a speculative working model unless direct biochemical or genetic evidence is provided.

Extracellular vesicles (EVs), particularly exosomes, provide another layer of communication by transferring proteins, lipids, and nucleic acids from TAMs to tumor cells (85–87). These mechanisms may contribute to therapy resistance and phenotypic remodeling, but their precise role in DNPC-associated lineage plasticity remains to be defined.

### 3.2 Established TAM-derived pathways and emerging candidate mediators

TNFAIP8L2 (TIPE2) was originally characterized as an immune regulatory molecule involved in maintaining immune homeostasis (60). It has subsequently been implicated in tumor biology and myeloid-cell function (51, 61, 62), but the receptor identity, context-specific signaling partners, and direct relevance of TNFAIP8L2 to prostate cancer lineage plasticity remain incompletely defined. PLAC8+ TAMs have been reported to express TNFAIP8L2 after ADT/ARSI exposure, and spatial transcriptomic analyses have placed PLAC8+ TAMs near CD8+ TRM-like cells and DNPC-like tumor regions (11). A conservative working model is that TNFAIP8L2-associated signaling may influence immune-cell function and may intersect with tumor-cell plasticity pathways such as PI3K/Akt, beta-catenin, AP-1, and FOSL1-HMGA1. At present, this should be presented as a hypothesis-generating model rather than a validated signaling cascade.

#### 3.2.1 IL-6/STAT3 signaling in stemness and neuroendocrine differentiation

Interleukin-6 (IL-6) is a TAM-derived factor with more established links to prostate cancer plasticity and treatment resistance. Produced by multiple stromal and immune cell types, IL-6 activates STAT3 signaling in tumor cells, leading to transcriptional upregulation of stemness-associated factors such as SOX2, NANOG, and OCT4 (68, 88, 89). Macrophage-derived secreted factors, including IL-6, can upregulate cancer stem-cell plasticity markers while suppressing AR and PSA, thereby creating a permissive state for lineage switching (69). In the context of neuroendocrine differentiation, IL-6/STAT3 signaling has also been implicated in the emergence of AR-low/NE-high tumor cell populations, although direct assignment of this effect to a specific TAM subset remains an area for further investigation (70).

#### 3.2.2 TGF-β as a context-dependent regulator of lineage identity

Transforming growth factor-beta (TGF-β), abundantly produced by multiple TAM and stromal populations, exerts complex and context-dependent effects on prostate cancer cells (71, 72). In epithelial tumor cells, TGF-β signaling through SMAD transcription factors can promote epithelial-mesenchymal transition, stem-like programs, and resistance-associated phenotypes. TGF-beta can also suppress CD8+ T-cell function and contribute to immune exclusion while influencing lineage plasticity. Within proposed triadic niches, TAM- or stromal-derived TGF-beta should therefore be considered a plausible component of a broader immunosuppressive and pro-plasticity milieu, rather than a fully resolved causal mechanism.

#### 3.2.3 Additional secreted mediators and coordinated spatial niches

Additional mediators may further connect macrophage, stromal, and tumor-cell states. IL-10 can reinforce immune suppression, while CXCL12 produced by iCAFs can recruit CXCR4-expressing immune or tumor cells (67). In proposed triadic aggregates, CXCL12+ iCAFs may spatially position lymphocytes near macrophage-rich regions. This model is plausible but remains correlative unless validated by perturbing the relevant chemokine axes in spatially preserved systems.

#### 3.2.4 Parallel mechanisms in SPP1^hi^-TAMs: adenosine signaling

While the factors discussed above are primarily associated with PLAC8^+^ TAMs and related subsets, independent investigations have revealed that SPP1hi-TAMs employ distinct molecular mechanisms to achieve immunosuppressive endpoints. Pathway analysis of SPP1hi-TAMs identified significant activation of hypoxia pathways, which promote extracellular adenosine accumulation through upregulation of CD39 and CD73. Engagement of adenosine A2A receptors (A2ARs) on T cells initiates downstream immunosuppressive signaling via intracellular cAMP, reducing the antitumor activity of CD8^+^ T cells and NK cells (7). In prostate cancer models, A2AR pathway inhibition has been linked to reversal of SPP1hi-TAM-associated T-cell suppression and improved responsiveness to PD-1 blockade, whereas clinical experience with A2AR blockade in other solid tumors provides a broader translational context rather than prostate cancer-specific proof (7, 63, 73). These findings illustrate that distinct TAM subsets—PLAC8^+^ and SPP1hi—may employ parallel but mechanistically distinct strategies to suppress anti-tumor immunity, with implications for developing subset-specific therapeutic interventions.

Multiple TAM-derived signals converge on pathways such as STAT3, NF-kappaB, PI3K/Akt, and beta-catenin. These networks may create permissive conditions for lineage plasticity, but the relative contribution of each macrophage subset and effector molecule remains unresolved. Therefore, subset-specific mediators such as TNFAIP8L2 should be considered candidate nodes within a broader signaling network.

### 3.3 Speculative mechanisms under ARSI pressure: P450 pathways and future hypotheses

The enrichment of cytochrome P450 (CYP) or xenobiotic metabolism signatures in PLAC8+ TAMs is intriguing, but its functional significance in prostate cancer remains speculative (90, 91). Possible mechanisms include altered local drug metabolism, production of lipid or steroid-like metabolites, and paracrine signaling that indirectly supports AR bypass (92, 93). These possibilities should not be presented as established ARSI-resistance mechanisms. Instead, they define future hypotheses that require spatial metabolomics, macrophage-specific perturbation, and biochemical assays to determine whether PLAC8+ TAMs alter intratumoral drug or lipid metabolism in a clinically meaningful way.

### 3.4 Tumor-cell intrinsic plasticity modules downstream of microenvironmental cues

TAM-derived signals may converge on tumor-cell pathways that regulate lineage identity, including STAT3, NF-kappaB, PI3K/Akt, beta-catenin, and AP-1-related transcriptional programs (66, 88, 94–97). The beta-catenin/AP-1/FOSL1 axis has been implicated in transcriptional reprogramming and basal-like states (95, 96), providing a plausible tumor-cell-intrinsic framework through which inflammatory or stromal signals might influence plasticity. However, direct upstream control of this axis by PLAC8+ TAM-derived TNFAIP8L2 remains unresolved.

**Figure 2:**
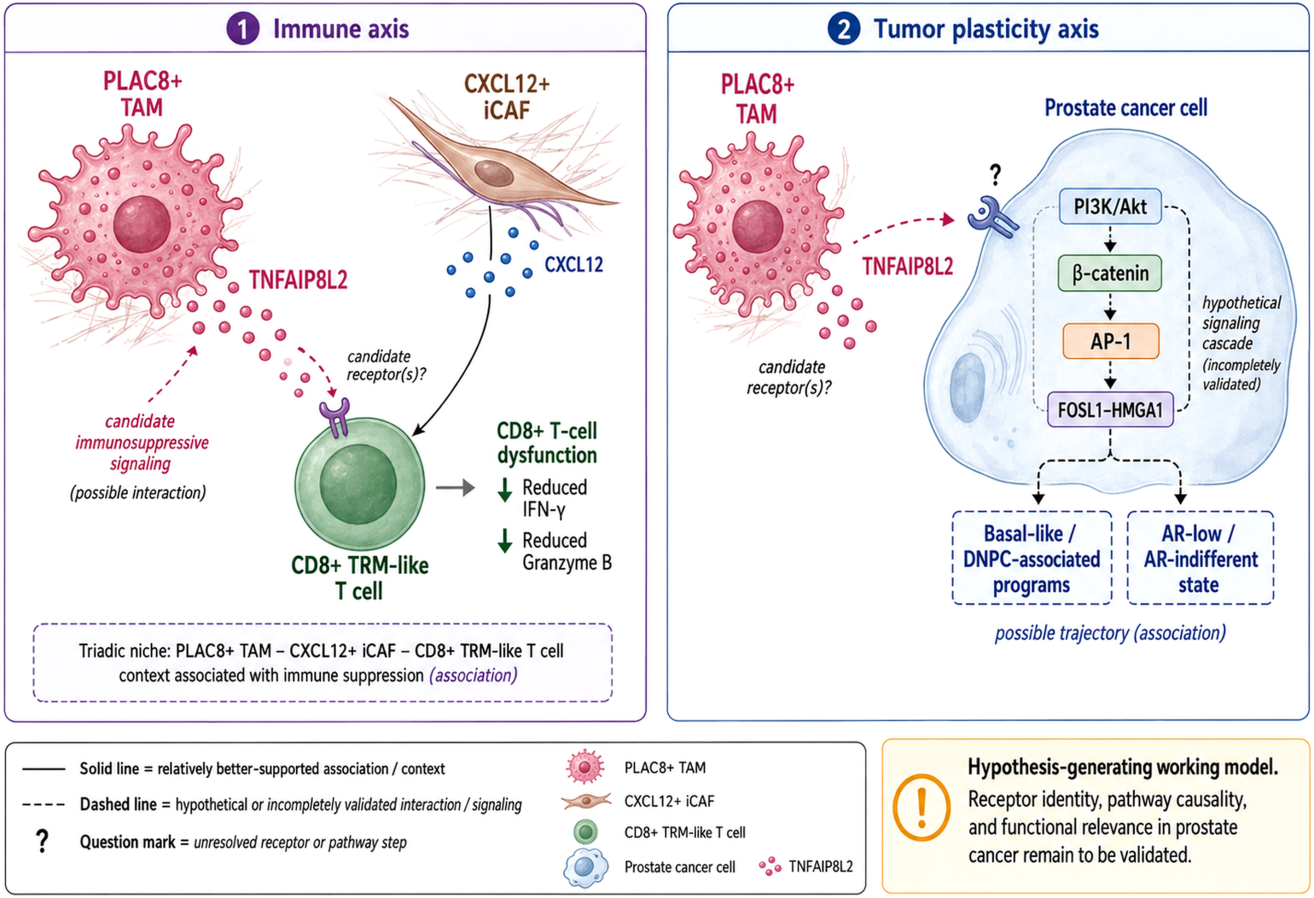
Working model of PLAC8+ TAM-associated immune suppression and lineage plasticity in advanced prostate cancer. The figure presents a hypothesis-generating working model divided into an immune axis and a tumor plasticity axis. In the immune axis, PLAC8+ TAMs may express TNFAIP8L2 within a CXCL12+ iCAF/CD8+ TRM-like T-cell niche, potentially contributing to reduced IFN-gamma and granzyme B expression through candidate immunosuppressive signaling. In the tumor plasticity axis, TNFAIP8L2-associated signaling is shown as a possible link to PI3K/Akt, beta-catenin, AP-1, and FOSL1-HMGA1 modules, which may intersect with basal-like or AR-indifferent programs. Solid lines denote relatively better-supported context, dashed lines denote hypothetical or incompletely validated interactions, and question marks indicate unresolved receptors or pathway steps. Receptor identity, pathway causality, and functional relevance in prostate cancer remain to be validated.

The FOSL1-HMGA1 axis can promote basal-lineage programs, including expression of KRT5, KRT14, TP63, and EMT-related regulators, while reducing luminal or AR-dependent features. In parallel, NF-kappaB activated by TAM-derived inflammatory factors can regulate stemness-associated factors and cooperate with STAT3-dependent cytokine circuits (97). Together, these pathways support the concept that macrophage-derived cues can create a permissive signaling environment for lineage plasticity, while leaving open the question of which TAM subsets and effector molecules are necessary in prostate cancer. This hypothesis-generating framework is summarized in **Figure 2**.

### 3.5 Epigenetic reprogramming downstream of TAM signals

TAM-associated inflammatory and suppressive cues may intersect with epigenetic regulators of lineage identity, including EZH2, HDACs, DNMTs, and non-coding RNA programs (98–103). These mechanisms provide a plausible route through which transient microenvironmental cues could become stabilized as durable transcriptional states. Integrative single-cell, spatial, and machine-learning approaches can further nominate candidate molecular nodes associated with aggressive prostate cancer, such as PCSK1N and chemokine-enriched gland signatures (67, 104). Such candidates should be viewed as priorities for validation rather than as established TAM-regulated effectors.

#### 3.5.1 Summary of part II

In summary, TAMs may influence prostate cancer lineage plasticity through a network of cytokines, chemokines, extracellular vesicles, metabolic cues, and epigenetic regulators. IL-6/STAT3, TGF-beta, NF-kappaB, CXCL12/CXCR4, and adenosine signaling currently provide a more established foundation than a single TNFAIP8L2-centered mechanism. TNFAIP8L2 remains an important emerging candidate, but the proposed TNFAIP8L2-integrin/beta-catenin/FOSL1-HMGA1 pathway should be regarded as a testable working model requiring direct validation.

## 4 Spatial organization of TAM-stromal-tumor crosstalk revealed by spatial omics

**Figure 3:**
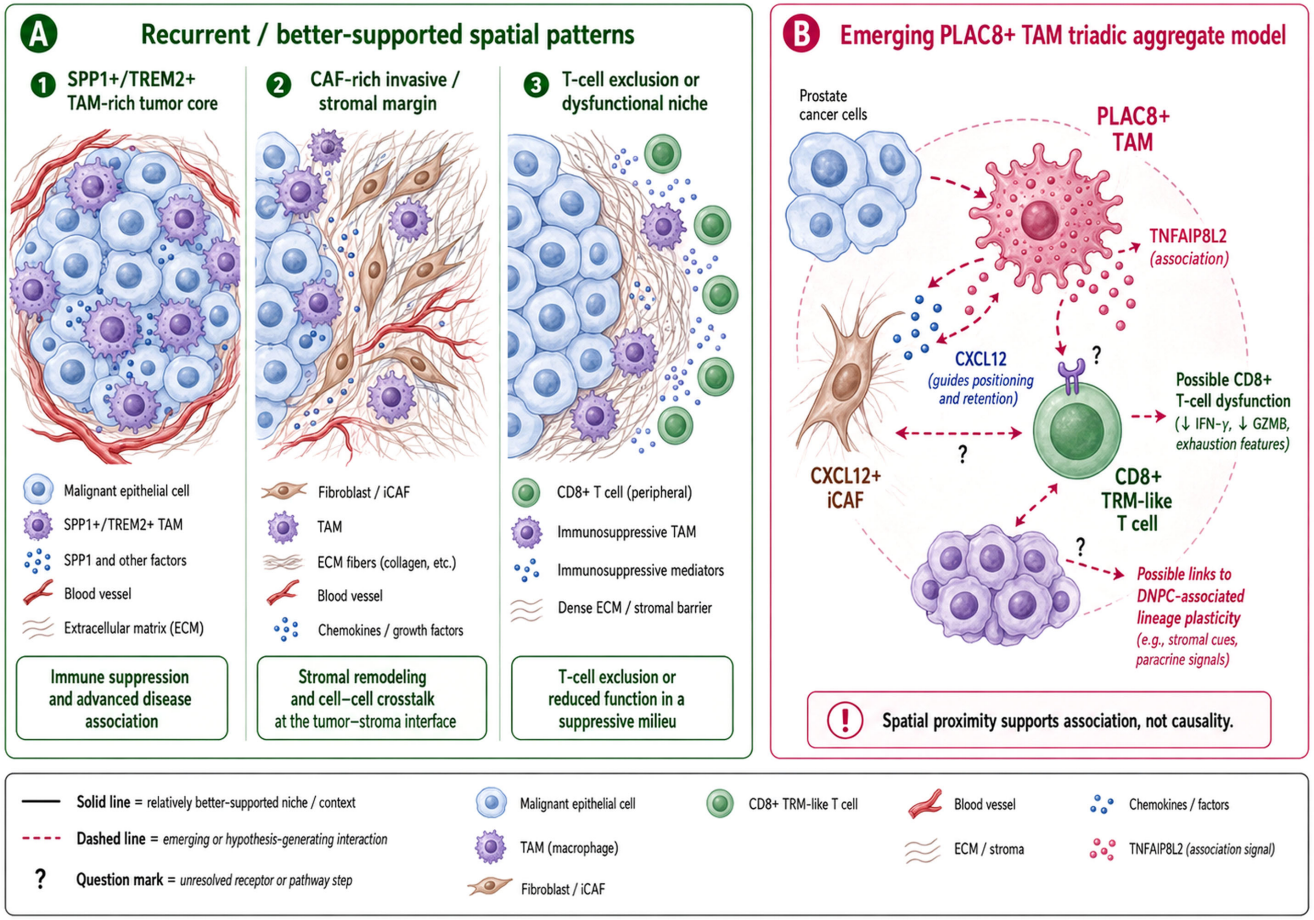
Spatial immune niches in prostate cancer: established patterns and emerging triadic aggregates. **(A)** summarizes recurrent and better-supported spatial patterns in prostate cancer, including SPP1+/TREM2+ TAM-rich tumor regions, CAF-rich invasive or stromal margins, and T-cell exclusion or dysfunctional niches. **(B)** depicts the emerging PLAC8+ TAM/CXCL12+ iCAF/CD8+ TRM-like T-cell triadic aggregate model. Dashed arrows indicate incompletely validated interactions, including TNFAIP8L2-associated signaling, CXCL12-guided positioning, possible CD8+ T-cell dysfunction, and possible links to DNPC-associated plasticity. The figure explicitly distinguishes spatial association from causality.

Spatial transcriptomic and proteomic technologies provide essential information about how TAMs, stromal cells, lymphocytes, and tumor cells are arranged in intact tissue (20). These approaches can identify non-random neighborhoods and generate mechanistic hypotheses, but they cannot alone prove causal interactions. This limitation is central when interpreting proposed multicellular structures such as triadic aggregates. **Figure 3** contrasts recurrent spatial patterns with the emerging PLAC8+ TAM triadic aggregate model.

### 4.1 Evolution of spatial transcriptomic technologies

Spatial transcriptomic and proteomic technologies now range from spot-based platforms such as 10x Genomics Visium to higher-resolution transcriptomic and protein imaging approaches, including Visium HD, MERFISH, seqFISH, CosMx SMI, PhenoCycler/CODEX, MIBI, and IMC (105–108). These platforms can map tissue neighborhoods and prioritize candidate interactions, but they differ substantially in resolution, target breadth, sensitivity, and protein-level validation capacity.

### 4.2 Spatial architecture of the prostate tumor microenvironment

Application of spatial transcriptomic and proteomic technologies to prostate cancer has begun to reveal the tissue architecture of the tumor microenvironment and its remodeling during progression and therapy (109–112). In normal prostate tissue, epithelial cells are organized into glandular structures surrounded by stromal compartments containing fibroblasts, smooth muscle cells, and scattered resident immune cells. In cancer, spatial analyses identify tumor cores, invasive margins, metastatic niches, and adjacent stromal regions with distinct cellular compositions and molecular programs. TAM localization is not random: SPP1+ TAMs have been associated with hypoxic regions, invasive fronts, metastatic niches, and stromal-remodeling programs in prostate cancer-specific single-cell or spatial studies (7, 30, 47, 110).

Mouse and human spatial atlases provide useful experimental systems for tracking therapy-associated epithelial and immune remodeling, although cross-species conservation should be interpreted cautiously (23, 24, 32). These datasets are particularly valuable for generating spatial hypotheses, defining candidate cell neighborhoods, and selecting markers for protein-level validation, but they do not by themselves establish the directionality or necessity of cell-cell communication.

### 4.3 The PLAC8+ TAM/CXCL12+ iCAF/CD8+ TRM triadic aggregate as an emerging spatial model

The triadic spatial aggregate is a recently proposed multicellular configuration involving PLAC8+ TAMs, CXCL12+ inflammatory cancer-associated fibroblasts (iCAFs), and CD8+ tissue-resident memory-like T cells (11). Reported spatial analyses suggest that CXCL12+ iCAFs may occupy peripheral positions, CD8+ TRM-like cells may localize near stromal and myeloid elements, and PLAC8+ TAMs may be enriched in the same neighborhood. This configuration provides a useful spatial framework for considering how stromal recruitment cues, myeloid immunoregulation, and tumor-cell plasticity may coexist within the same tissue region.

Because spatial colocalization is correlative, TNFAIP8L2 expression in PLAC8+ TAMs should not be interpreted as proof that TNFAIP8L2 simultaneously acts on CD8+ TRMs and adjacent tumor cells. The clinical significance of triadic aggregates should also be interpreted cautiously. Associations between aggregate density, basal-like differentiation markers, ARSI-treated states, and immune dysfunction are hypothesis-generating and require independent cohorts, protein-level validation, and perturbation experiments. Other prostate cancer spatial and single-cell studies support non-random myeloid, stromal, epithelial, and metastatic niche organization, including SPP1+ TAM-rich regions, CAF-associated tumor margins, and immune-excluded neighborhoods; these broader spatial patterns provide an important counterbalance to over-focusing on a single PLAC8+ TAM-centered model (7, 23, 30, 32, 110, 112–114).

### 4.4 Functional implications of spatial organization

Spatial organization is useful for generating mechanistic and therapeutic hypotheses, but interpretation must remain conservative: proximity does not prove ligand-receptor signaling, transcript abundance does not prove protein secretion, and neighborhood correlations do not prove functional dependence. Future studies should combine spatial transcriptomics with multiplexed protein imaging, RNAscope or spatial metabolomics (115), ex vivo co-culture, macrophage-specific perturbation, and *in vivo* therapeutic models to test whether candidate niches actually regulate tumor-cell state or immune function.

### 4.5 Summary of part III

In summary, spatial technologies have revealed non-random TAM organization in prostate cancer and generated important hypotheses about TAM-stromal-tumor crosstalk. The PLAC8+ TAM/CXCL12+ iCAF/CD8+ TRM triadic aggregate is a promising emerging model, but its causal role in immune suppression, ARSI resistance, and DNPC plasticity remains to be established by protein-level validation and functional perturbation.

## 5 Therapeutic implications of TAM-associated plasticity

TAM-directed therapies are conceptually attractive because macrophages can influence immune suppression, stromal remodeling, and therapy resistance. However, therapeutic claims must be proportional to evidence maturity. An evidence-aware summary of TAM-directed and microenvironment-directed therapeutic strategies, their current evidence maturity, and key caveats is provided in **Table 2**. Current approaches can be broadly categorized as depletion, reprogramming, and functional blockade, and may be combined with ARSIs or immune checkpoint blockade in carefully selected contexts (116–118, 131, 132). Broad CSF1R-directed depletion has shown limited activity and potential toxicity, whereas reprogramming strategies such as CD40 agonism, TLR agonism, PI3K-gamma inhibition, or adenosine/A2AR blockade may be more context-dependent (75–83, 121–124).

**Table 2:** Evidence-aware TAM-directed and microenvironment-directed therapeutic strategies in prostate cancer.

Strategy	Targeted program	Evidence maturity	Proposed use and caveats	Representative references
HLA-DQB2-directed depletion	PLAC8+ TAM-like cells	Conceptual/preclinical	Test whether HLA-DQB2 can identify and deplete treatment-associated TAMs; requires independent protein validation, specificity, safety and efficacy data.	(11, 119, 120)
TNFAIP8L2 neutralization	PLAC8+ TAM candidate effector	Hypothesis-generating	Perturb TNFAIP8L2 to test effects on T cells and tumor-cell plasticity; receptor identity, secretion, pathway causality and normal-tissue function remain unresolved.	(51, 52, 60–62)
A2AR/adenosine blockade	SPP1hi or hypoxia-associated TAM programs	Preclinical and early clinical rationale	Reverse adenosine-mediated immunosuppression and combine with ICB in selected contexts; requires PCa-specific biomarker stratification.	(7, 63, 73)
CSF1R pathway inhibition	Broad macrophage programs	Clinical testing; modest activity	Reduce macrophage survival/recruitment or combine with other agents; limited subset specificity, toxicity and compensatory myeloid expansion are concerns.	(49, 75–77)
PI3K-gamma/CD40 reprogramming	Suppressive myeloid states	Preclinical/early clinical	Shift macrophage phenotypes toward antigen-presenting or inflammatory programs; M1/M2 terminology should be used cautiously and biomarkers are needed.	(78, 121–124)
CXCL12/CXCR4 spatial disruption	CAF-immune niches	Clinical/preclinical across tumors	Alter stromal-immune organization and immune exclusion; selective disruption of PLAC8+ triadic aggregates remains unproven.	(67, 125, 126)
ARSI plus immunotherapy combinations	Tumor-cell AR, T cells and antigen presentation	Mechanistically supported; clinical context-dependent	Leverage AR blockade to improve CD8+ T-cell function and MHC class I antigen presentation; benefit in unselected mCRPC remains limited.	(7–9)

### 5.1 Overview of TAM-targeting strategies

### 5.2 Subset-specific strategies: evidence maturity matters

PLAC8+ TAMs, HLA-DQB2, and TNFAIP8L2 provide an intriguing basis for subset-specific therapeutic hypotheses, but these strategies remain at an early stage. The revised manuscript therefore describes HLA-DQB2-directed depletion and TNFAIP8L2 neutralization as proposed or preclinical concepts rather than established precision therapies for DNPC.

HLA-DQB2-directed cell depletion. HLA-DQB2 has been proposed as a candidate surface marker for PLAC8+ TAMs (119, 120). Before therapeutic development can be prioritized, its specificity, protein-level expression, safety profile, and functional relevance must be validated across independent prostate cancer cohorts and *in vivo* models.

TNFAIP8L2 neutralization. TNFAIP8L2 is an emerging candidate effector associated with PLAC8+ TAMs, but it should not yet be described as a master mediator of both immune suppression and tumor plasticity. Neutralization strategies are best presented as hypothesis-driven approaches requiring receptor identification, pharmacodynamic biomarkers, and functional testing in ARSI-treated prostate cancer models.

Disrupting the triadic aggregate. CXCL12/CXCR4 inhibition and spatial niche disruption are plausible strategies for altering stromal-immune organization (127). However, whether disrupting this axis selectively affects PLAC8+ TAM-containing aggregates or improves ARSI/ICB response in prostate cancer remains to be demonstrated.

### 5.3 Rational combination strategies

The heterogeneous roles of TAMs support rational combination strategies, but these should be framed as testable therapeutic hypotheses rather than inevitable clinical solutions. ARSI therapy may reshape macrophage states and create treatment-associated myeloid vulnerabilities (128, 129), while prostate cancer generally responds poorly to immune checkpoint blockade in unselected populations (8, 9, 130). In this setting, myeloid reprogramming or functional blockade might improve immune responsiveness only if the relevant suppressive program is present and targetable. SPP1+ TAMs provide a more independently supported rationale for TAM-directed combination strategies in prostate cancer (7), whereas PLAC8+ TAM/HLA-DQB2/TNFAIP8L2-directed strategies remain earlier-stage concepts.

Triple combination strategies combining TAM targeting, ARSI, and immunotherapy may eventually be relevant, but they require careful preclinical prioritization, toxicity assessment, and biomarker selection. Biomarker-guided selection will be essential because prostate cancer TAM states are spatially and transcriptionally heterogeneous (114). Future scoring systems should integrate TAM burden, TAM state, stromal architecture, epithelial lineage state, and T-cell function rather than relying on any single marker.

### 5.4 Clinical translation progress and challenges

Clinical translation of TAM-directed strategies in prostate cancer remains challenging because of uncertain target specificity, compensatory myeloid plasticity, limited efficacy of broad macrophage depletion, and the need for clinically deployable biomarkers. These issues are particularly substantial for PLAC8+ TAM targeting. Before HLA-DQB2 or TNFAIP8L2 can be prioritized therapeutically, independent studies must validate PLAC8+ TAM abundance, confirm protein-level expression, define receptor biology and causal pathway dependence, and demonstrate that intervention improves tumor or immune phenotypes without unacceptable toxicity.

### 5.5 Summary of part IV

In summary, TAM-directed therapy remains a promising but evidence-dependent frontier in prostate cancer. Strategies targeting SPP1+/TREM2+ programs, adenosine signaling, or broader TAM biology have different levels of support than PLAC8+/TNFAIP8L2-directed approaches. The revised manuscript therefore frames subset-specific PLAC8+ TAM targeting as a hypothesis-driven direction requiring independent validation, rather than as an established precision therapy for overcoming DNPC progression.

## 6 Validation roadmap, conclusions, and future perspectives

### 6.1 Synthesis of key insights and evidence levels

Single-cell and spatial technologies have transformed our understanding of TAM biology in prostate cancer, replacing a simplistic M1/M2 framework with a view of heterogeneous, spatially organized, and therapy-responsive macrophage states. The integrated evidence reviewed here supports several principles. First, TAM heterogeneity is functionally relevant, but evidence maturity differs by subset: SPP1+/TREM2+ macrophage programs are relatively well supported in advanced prostate cancer, whereas PLAC8+ TAMs remain an emerging treatment-associated state. Second, TAM functions extend beyond immune suppression and may influence tumor-cell phenotype through cytokines, chemokines, metabolic cues, and stromal interactions; among these, IL-6/STAT3, TGF-beta, adenosine, and NF-kappaB pathways currently have stronger mechanistic support than the TNFAIP8L2-centered model.

Third, TAM-tumor-stromal interactions are spatially organized, but spatial organization alone is not causal evidence. Triadic aggregates involving PLAC8+ TAMs, CXCL12+ iCAFs, and CD8+ TRM-like cells should be treated as an emerging spatial model. Fourth, TAM states may evolve under ADT and ARSI pressure, but the ontogeny, stability, and functional consequences of PLAC8+ TAM enrichment remain unresolved. Finally, integrative multi-omics and explainable AI can nominate candidate biomarkers and spatial signatures, but these candidates must be validated analytically, biologically, and clinically before they are used to guide therapy. Together, these findings support an ecosystem-level framework in which TAMs, tumor-cell lineage programs, stromal architecture, and immune suppression co-evolve during prostate cancer progression.

### 6.2 Outstanding questions

Despite substantial progress, translating spatial and molecular TAM insights into durable clinical interventions remains an early-stage challenge. The field must move from correlative atlases to experimentally tested mechanisms. Key unresolved questions include the ontogeny and developmental trajectories of PLAC8+, SPP1+, TREM2+, and other TAM states (133); the redundancy and specificity of effector mechanisms such as TNFAIP8L2, adenosine signaling, IL-6, and TGF-beta (134); the adaptive plasticity of TAM states after ARSI, immunotherapy, or TAM-directed intervention; the spatiotemporal dynamics of immune niches in static versus longitudinal datasets (135); and the possible field effects created by chemokine-enriched gland signatures and immune-stromal changes in histologically benign regions (136).

### 6.3 Technological and experimental validation roadmap

**Figure 4:**
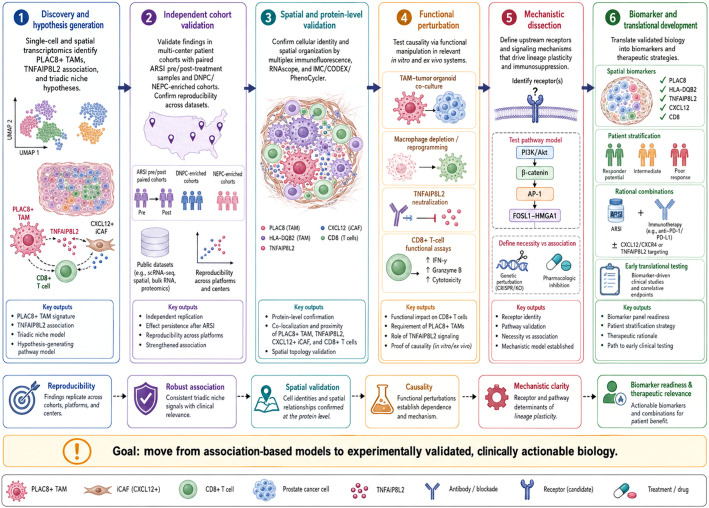
Validation roadmap for PLAC8+ TAM-associated lineage plasticity models. The roadmap illustrates a staged path from association-based discovery to clinically actionable biology. Step 1 uses single-cell and spatial transcriptomics to generate hypotheses about PLAC8+ TAMs, TNFAIP8L2, and triadic niches. Step 2 requires independent cohort validation, including paired ARSI pre/post-treatment samples and DNPC- or NEPC-enriched cohorts. Step 3 prioritizes spatial and protein-level validation using multiplex immunofluorescence, RNAscope, IMC/CODEX, or PhenoCycler. Step 4 tests causality through organoid co-culture, macrophage perturbation, TNFAIP8L2 neutralization, and CD8+ T-cell functional assays. Step 5 dissects receptor identity and signaling necessity, and Step 6 translates validated biology into biomarkers, patient stratification, rational combinations, and early translational studies.

The next stage of this field should prioritize validation rather than expansion of untested mechanisms. High-resolution multi-omics should be integrated with protein-level assays, spatial metabolomics, longitudinal sampling, lineage tracing, and functional perturbation within a staged validation framework (**Figure 4**). Spatial multi-omics can help determine whether transcript-defined TAM states correspond to protein-level phenotypes and functional signaling activity (137), while explainable AI and predictive algorithms should be treated as hypothesis-generating tools whose outputs require reproducibility testing, biological plausibility checks, and functional validation (104, 138).

4D spatiotemporal tracking, lineage tracing, *in vivo* imaging, patient-derived organoids, organotypic tumor spheroids, and autologous TAM co-culture systems can help determine whether TAM states precede, follow, or reinforce tumor-cell lineage transitions (139, 140). Neutralization or knockout of candidate macrophage-derived factors, including TNFAIP8L2, together with tumor-cell receptor perturbation and pathway inhibition, will be necessary to establish causality and therapeutic relevance.

### 6.4 Clinical opportunities and translational caution

The ultimate goal is to convert TAM-associated biology into clinically useful biomarkers and therapies, but this will require staged development: independent validation of TAM states, robust tissue assays, mechanistic testing, and prospective clinical correlation. Candidate biomarkers such as HLA-DQB2, PLAC8, TNFAIP8L2, SPP1/TREM2, PCSK1N, and spatial proximity metrics should not be used clinically until their reproducibility, specificity, and predictive value are established. Similarly, TNFAIP8L2-directed therapy requires clarification of receptor identity, on-target function, and safety before translational prioritization.

Sequential and combinatorial treatment paradigms should be designed around validated biology. Combining TAM targeting with ARSI or immune checkpoint blockade may be rational in selected contexts, but patient selection, timing, dosing, and compensatory resistance mechanisms will be critical. Integrative precision oncology frameworks may ultimately incorporate TAM burden, stromal organization, epithelial lineage state, and immune activity, but such frameworks should remain transparent about evidence maturity and should avoid presenting spatially inferred mechanisms as therapeutically proven.

### 6.5 Concluding remarks

TAMs are increasingly recognized as heterogeneous and spatially organized components of the prostate cancer ecosystem. The strongest current evidence supports recurrent myeloid programs such as SPP1+/TREM2+ TAMs and established inflammatory pathways including IL-6, TGF-beta, NF-kappaB, CXCL12/CXCR4, and adenosine signaling (7, 30, 47). PLAC8+ TAMs, TNFAIP8L2, and triadic spatial aggregates represent a compelling emerging framework for understanding ARSI-associated immune and lineage remodeling, but they remain incompletely validated and should be treated as hypothesis-generating rather than definitive mechanisms.

A balanced interpretation of this field requires distinguishing established mechanisms from emerging spatial associations and speculative therapeutic strategies. If validated through independent cohorts, protein-level spatial assays, and functional perturbation studies, TAM-associated biomarkers and targets may eventually guide rational combinations of ARSI, immunotherapy, and myeloid-directed intervention for treatment-resistant prostate cancer.

## Supplementary material

Supplementary Table S1 Detailed markers, biological context, evidence maturity, and representative sources for TAM-, myeloid-, and stromal programs discussed in prostate cancer.
