# Supplementary material for: Tumor-associated macrophages and lineage plasticity in prostate cancer: from established myeloid programs to emerging spatial hypotheses
Source: Front Immunol. 2026 Jul 13;17:1853196. doi: 10.3389/fimmu.2026.1853196 (PMC13402378; doi:10.3389/fimmu.2026.1853196)
Supplement: Supplementary Table S1 — Detailed markers, biological context, evidence maturity, and representative sources for TAM-, myeloid-, and stromal programs discussed in prostate cancer. [file Table1.docx]

**Supplementary Table S1. Detailed TAM and myeloid/stromal programs discussed in the review**

**Note.** This supplementary table expands the evidence hierarchy summarized in the main manuscript. Marker sets are state-associated rather than lineage-defining; evidence levels reflect the current prostate cancer literature and should be updated as independent cohorts and functional studies become available.

| Program / state | Typical context in prostate cancer | Representative markers / features | Candidate mediators or functions | Evidence maturity and interpretation | Representative refs |
| --- | --- | --- | --- | --- | --- |
| SPP1+/TREM2+ TAMs | Advanced, metastatic, hypoxic, stromal-remodeling or immunosuppressive regions | SPP1, TREM2, APOE, C1QC/C1QA, CD163/CD206, MERTK; often enriched in myeloid suppressive programs | ECM remodeling, angiogenesis, antigen-presentation impairment, adenosine-related immune suppression, T-cell dysfunction | Relatively well supported by multiple prostate cancer single-cell/spatial studies; subset definition and functional dependence still require context-specific validation | [7,30,47,48,63] |
| PLAC8+ TAMs | Treatment-associated / ARSI-exposed disease; proposed association with DNPC-like or basal-like programs | PLAC8, TNFAIP8L2, MARCO/CD204; candidate HLA-DQB2 surface marker; possible xenobiotic/metabolic signatures | Candidate TNFAIP8L2-associated immune modulation and possible links to tumor plasticity; receptor identity unresolved | Emerging prostate cancer-specific observation; should be treated as hypothesis-generating until independently validated at protein and functional levels | [11,51,52,60-62] |
| CSF1R+/CD163+ macrophage programs | Broad macrophage populations in primary and advanced prostate tumors; variable association with ADT/ARSI response | CSF1R, CD68, CD163, CD206, MRC1; partial overlap with M2-like annotations | Macrophage survival, tumor support, immune suppression, resistance to androgen blockade in selected models | Established as a macrophage axis, but broad depletion has limited specificity and variable translational efficacy | [33,49,75-77] |
| APOE+/C1QC+ resident-like TAMs | Resident or tissue-adapted macrophage states; may vary by disease stage, age, and tissue niche | APOE, C1QC/C1QA, complement genes, phagocytic and lipid-handling programs | Phagocytosis, lipid metabolism, immune regulation, tissue remodeling | Recurrent in single-cell atlases, but prostate cancer-specific functions remain incompletely defined | [16,23,26,35,36] |
| Hypoxia/adenosine-associated myeloid program | Advanced tumors and suppressive niches, including SPP1hi macrophage-associated contexts | CD39/ENTPD1, CD73/NT5E-related pathways, hypoxia signatures, A2AR axis | Extracellular adenosine accumulation, suppression of CD8+ T cells and NK cells, potential checkpoint blockade resistance | Mechanistically supported in cancer immunology; prostate cancer-specific application is promising but still evolving | [7,63,73] |
| IL-6/STAT3-associated TAM/stromal signaling | Inflammatory and therapy-resistant prostate tumor microenvironments | IL6, STAT3 activation, NANOG/SOX2/OCT4/CD44-associated stemness programs | Stemness, AR suppression, neuroendocrine or AR-low plasticity, immune evasion | Relatively established pathway-level evidence; cell-state-specific contribution of individual TAM subsets remains unresolved | [68-70,88,89] |
| TGF-beta-associated suppressive/stromal signaling | CAF-rich, immune-excluded, or EMT-associated microenvironments | TGFB1/2/3, SMAD targets, EMT markers, stromal remodeling genes | EMT, immune exclusion, CD8+ T-cell suppression, stromal barrier formation | Established pathway in cancer immunity; contribution to specific PLAC8+ triadic niches remains hypothetical | [71,72,113,114] |
| CXCL12+ iCAF / stromal niche | Stromal margins and proposed triadic aggregate contexts | CXCL12, CAF/iCAF markers, ECM and chemokine programs | Chemotaxis, immune-cell positioning or retention, stromal-immune organization | Supported as a recurrent stromal program; role in PLAC8+ TAM/CD8+ TRM triads is emerging and spatial-association based | [11,112,125-127] |
| PMN-MDSC / compensatory suppressive myeloid states | Inflamed or therapy-adapted tumors; potential compensation after TAM targeting | CD11b, CD15, LOX-1, ARG1, ROS/peroxynitrite programs | T-cell suppression, compensatory immune evasion, resistance to single-agent myeloid targeting | Conceptually supported in tumor immunology; prostate cancer-specific integration with TAM plasticity requires more data | [26,33,74,134] |
| Triadic aggregate model | Proposed PLAC8+ TAM/CXCL12+ iCAF/CD8+ TRM-like niche near DNPC-associated tumor regions | PLAC8+ TAMs, TNFAIP8L2 association, CXCL12+ iCAFs, CD8+ TRM-like cells, possible reduced IFN-gamma/GZMB | Spatial immune suppression and possible association with DNPC-like plasticity | Emerging spatial model; proximity supports association but not causality; requires independent cohorts and functional perturbation | [11,20-22,115] |

**Abbreviations:** ARSI, androgen receptor signaling inhibitor; CAF, cancer-associated fibroblast; DNPC, double-negative prostate cancer; ECM, extracellular matrix; iCAF, inflammatory CAF; PMN-MDSC, polymorphonuclear myeloid-derived suppressor cell; TAM, tumor-associated macrophage; TRM, tissue-resident memory T cell.
